# Focal invasiveness in complete histological analyses of a large acral lentiginous melanoma

**DOI:** 10.1186/s13000-015-0307-z

**Published:** 2015-06-20

**Authors:** José Cândido Caldeira Xavier-Júnior, Tania Munhoz, Vinicius Souza, Eloísa Bueno Pires de Campos, Hamilton Ometto Stolf, Mariângela Esther Alencar Marques

**Affiliations:** Department of Pathology, Botucatu Medical School, Paulista State University (UNESP), Rubião Júnior s/n 18618-970, Botucatu, SP Brazil; UNESP- Universidade Estadual Paulista Radiology and Dermatology Department, Botucatu, SP Brazil

**Keywords:** Melanoma, Dermatopathology, Cancer, Acral Lentiginous Melanoma

## Abstract

**Background:**

Acral lentiginous melanoma is a melanoma with poor prognosis which is frequently diagnosed at an advanced stage. Since the thickness of tumour is one of the main prognostic factors, this case can exemplify how important complete histological analyses looking for focal invasiveness can be.

**Case report:**

A 77 year-old woman with a black spot with slow progressive growth on the left plantar region. She sought medical attention due to the expansion onto the dorsal surface of toes. The lesion had irregular borders and had spread to half the plantar surface. Histopathology confirmed the clinical suspicion of acral lentiginous melanoma Clark level IV and 2.6 mm Breslow thickness. The surgical specimen was entirely processed for histological evaluation, requiring 53 slides. Tumor dermal invasion was detected in only three out of 53 glass slides as the invasiveness was not identified by clinical, dermatoscopy or macroscopy exams.

**Conclusion:**

Sectioning through the entire lesion is considered very important to determinate the appropriate stage of the disease and the correct treatment and patient follow-up.

**Virtual slides:**

The virtual slide(s) for this article can be found here: http://www.diagnosticpathology.diagnomx.eu/vs/1513617994148349.

## Background

Acral lentiginous melanoma (ALM) is a common type of melanoma which should be considered as a clinicopathologic entity [1]. AML is the most frequent type of melanoma in the Asian population and those with darker skin types [2]. Nevertheless, among the white population, there is a predominance of ALM in women, with a peak of incidence during the seventh decade and the foot is the most common site of ALM [3].

ALM occurs, as the name implies, on the acral skin surfaces, with plantar and subungual lesions being the most common sites in 60 % of patients [2]. It usually presents as a pigmented macule or papule with irregular borders and variegated pigmentation on the palmar and plantar regions [2]. It has been theorized that chronic trauma may be a predisposing factor [2], but the pathogenesis remains unknown [3].

A worse prognosis for ALM is observed than for other subtypes of melanoma. This may be partially explained by the following: patients with ALM are usually older than patients with other subtypes of melanoma; ALM is often diagnosed at a more advanced stage; the anatomic site of disease (acral region) is hidden and it may have an unusual clinical presentation [2–4].

The hallmark of ALM is characterized by an asymmetric proliferation of continuous single melanocytes at the dermal-epidermal junction, made up of single melanocytes over nests. Development of nests and prominent pagetoid spread would appear later in the evolution of this kind of tumor [2]. The American Joint Committee on Cancer (AJCC) melanoma staging from 2009 indicates that for localized disease, the thickness, ulceration and mitotic rate are the strongest prognostic factors when evaluating the primary tumor.

Since AML is frequently diagnosed at an advanced stage, there is no consensus about grossing procedure and the thickness of tumor is one of the main prognostic factors. It is important to exemplify how important complete histological analyses can be when looking for focal invasiveness.

## Case presentation

**Fig. 1:**
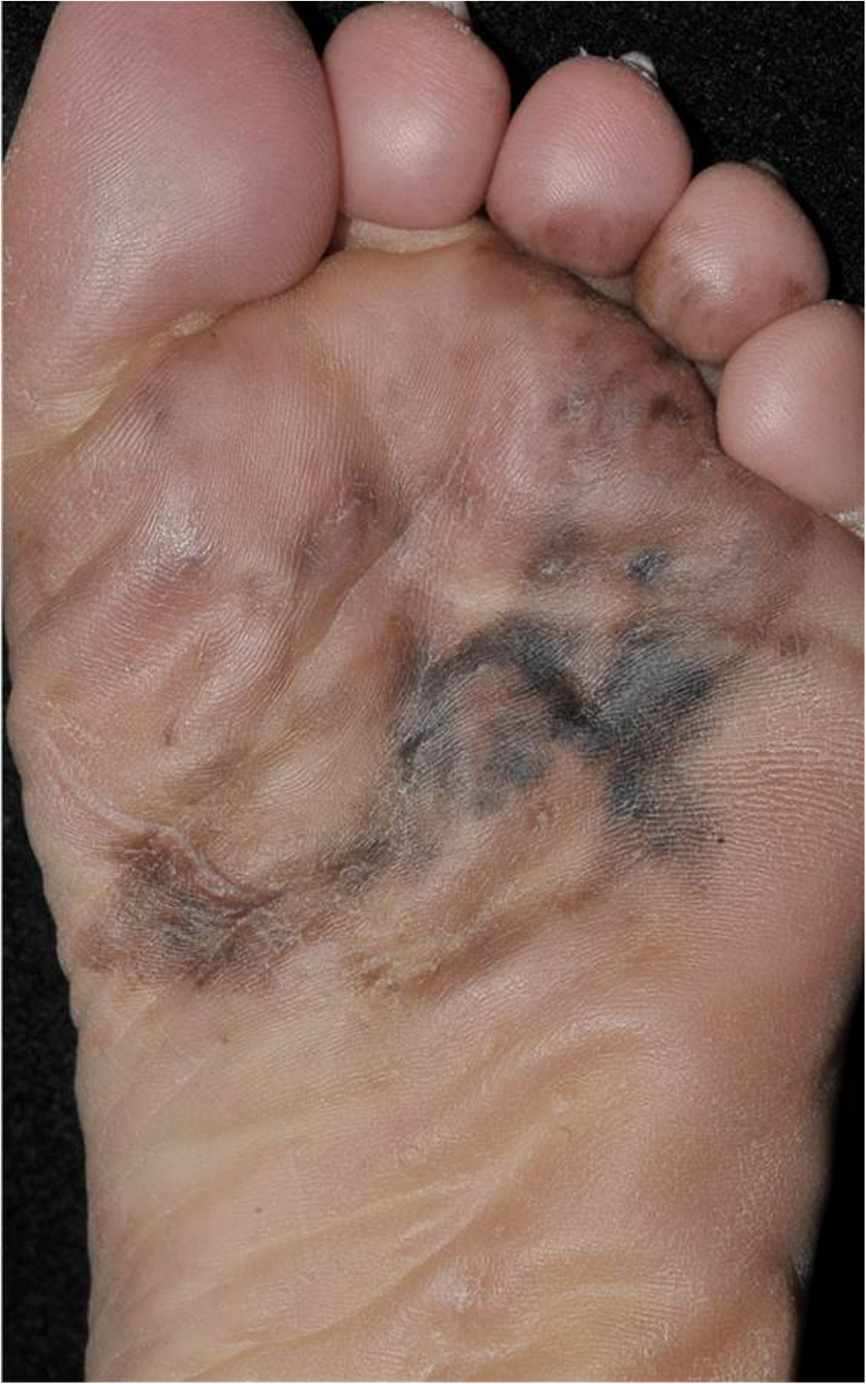
Large asymmetric dark-brown macule, irregular borders, with several different colors

**Fig. 2:**
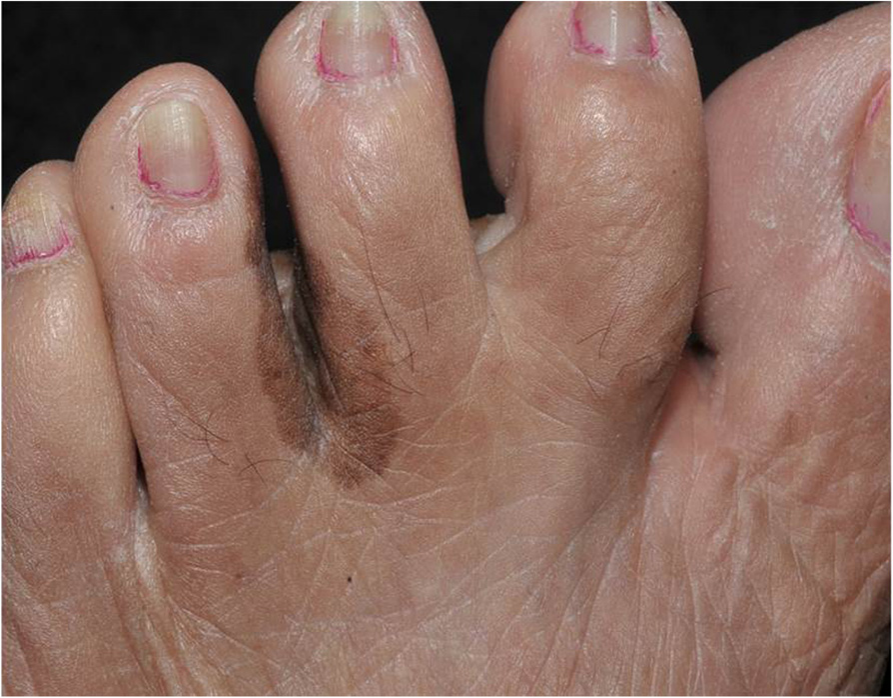
Brown macule progressing through interdigital region and dorsal surface of the fingers

A 77 year-old, woman presented with a black spot on the left plantar region which initially developed approximately 10 years ago, and had a slow progressive growth. She sought medical attention due to the lesion expanding onto the dorsal surface of toes. The lesion had irregular borders and spread to half plantar surface until the interdigital region (Figs. 1 and 2). She reported a previous history of ischemic stroke 8 years previously, type 2 diabetes mellitus, and hypertension.

In dermatoscopy analyses of darker areas, there was total blurring of the architectural pattern of the ridges and furrows, with obliteration of glandular ostia. In the lighter regions, there was a predilection of the pigment in the ridges. There was presence of peripheral rifling suggesting local expansion and growth; this was consistent with the clinical diagnosis of acral lentiginous melanoma. It is important to highlight that dermatoscopic analyses was not able to determinate focus of invasion in this case.

Surgical excision of the lesion and a sentinel lymph node biopsy was performed with a satisfactory start to the healing progress by second intention

The pathology specimen consisted of plantar skin measuring 9.5 × 9.0 × 0.5 cm, showing a macular lesion with irregular borders and with color variegation (brown, dark, black, gray) spreading to digit skin.

**Fig. 3:**
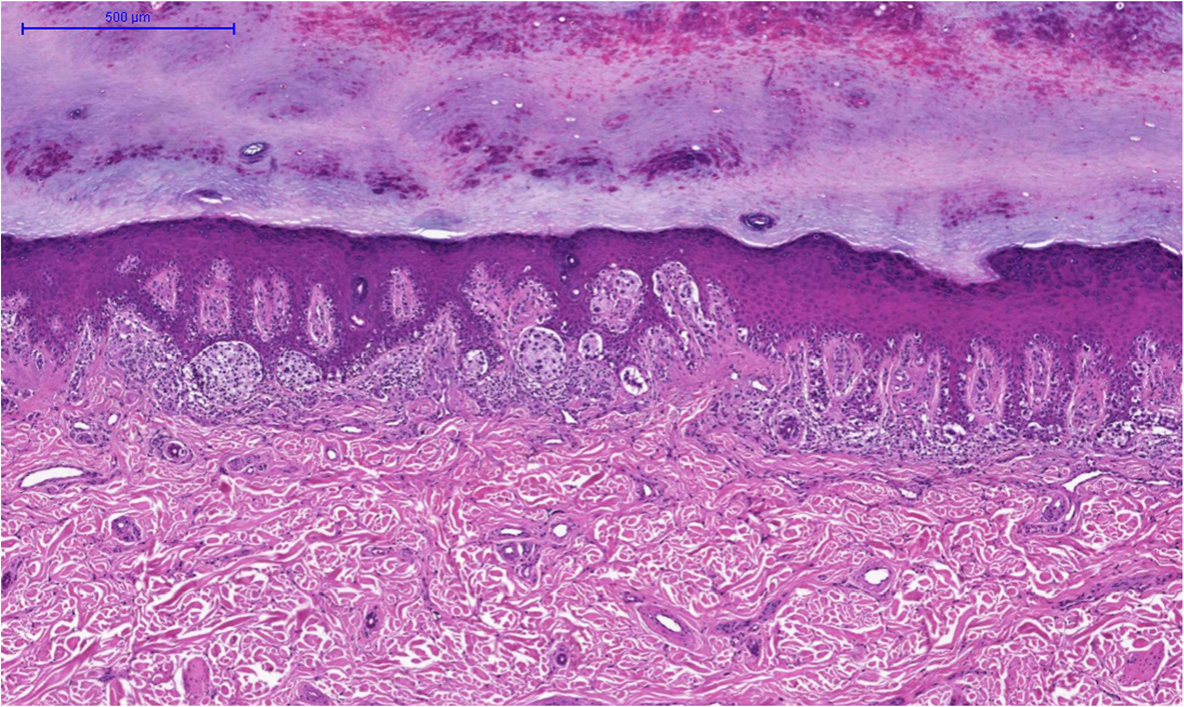
Acral lentiginous melanoma, “in situ” pattern. Most cells are arranged in a linear array along the basal layer and a few nests are also present HE

**Fig. 4:**
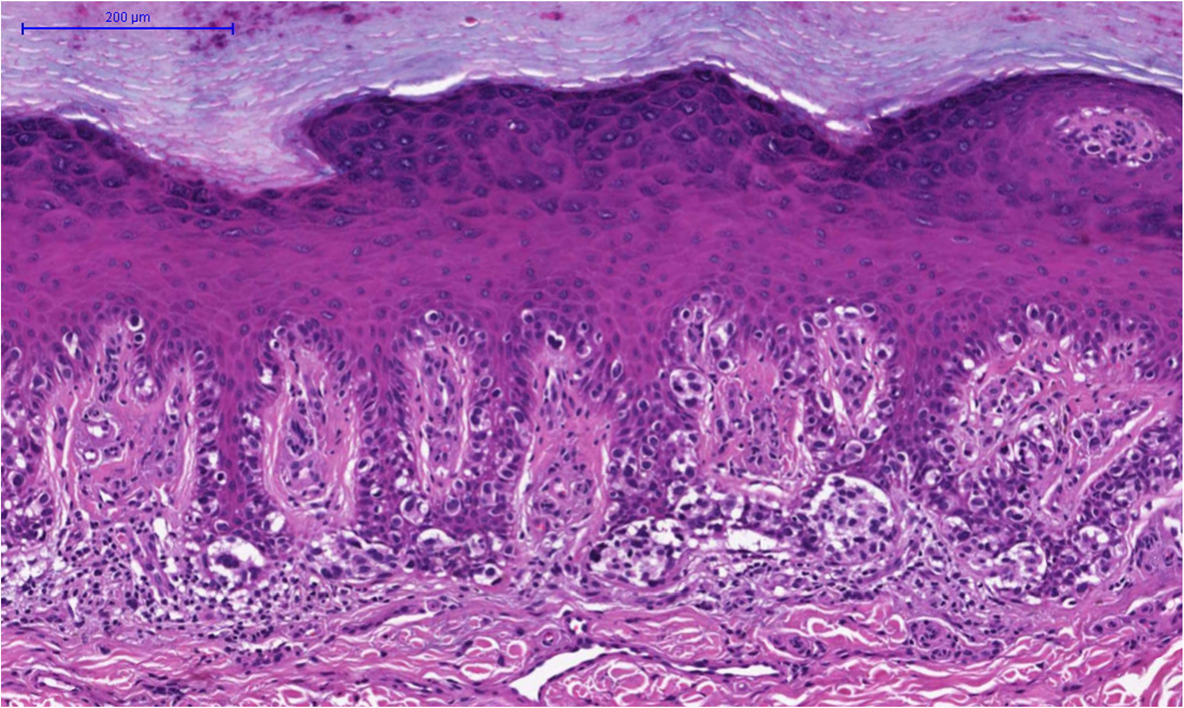
Acral lentiginous melanoma, “in situ” pattern, detail that points toward the lymphocytic infiltrate in the papillary dermis and the irregular shape of the nests HE

**Fig. 5:**
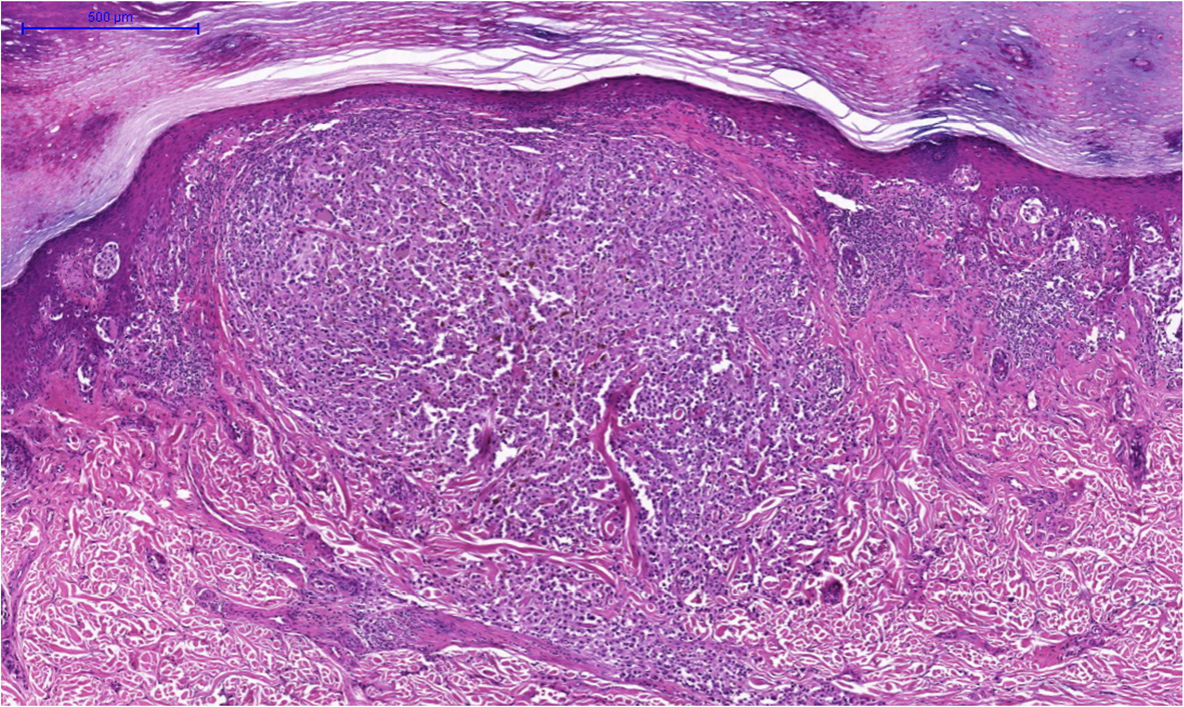
Acral lentiginous melanoma focal invasiveness showing a large expanding nodule infiltrating the reticular dermis HE

Histopathology confirmed the clinical suspicion of acral lentiginous melanoma, with a vertical growth phase of Clark level IV and 2.6 mm Breslow thickness. The mitotic index was 3 mitoses per mm^2^. There was no perineural or intravascular invasion but there was a mild inflammatory host response. There were areas of complete regression. There was no ulceration, microscopic satellites, associated nevus, or involvement of adnexal structures. The surgical margin of the hallux and the interdigital space between the third and fourth toes were positive with involvement by “in-situ” melanoma. (Figs. 3, 4 and 5) The other surgical margins were free from melanoma. The inguinal sentinel lymph node biopsy was negative for melanoma metastasis.

The surgical specimen was entirely sectioned for histological evaluation, requiring 53 slides. Tumor invasion was focal in only three slides, invading into the reticular dermis. The other slides demonstrated an increased density of large atypical melanocytes, characterized by nuclear pleomorphism and hyperchromasia and showing a cytoplasmic fixation retraction artifact, scattered along the basal layer and in junctional nests. The epidermis was atrophic with areas of pagetoid and the dermis showed prominent solar elastosis. It is necessary to highlight that the invasiveness had not been identified by clinical, dermatoscopy or macroscopy exams.

After multidisciplinary discussion and consideration of the positive surgical margins, transmetatarsal amputation with reconstruction using a skin graft from the right lower side of the abdomenal region was required. There was in-situ acral lentiginous melanoma in the residual skin, the surgical margins were free from melanoma.

The melanoma was stage IIA (T3a N0 M0) based on the AJCC staging scale.

The patient attends quarterly clinical follow-up with no evidence of melanoma recurrence with appreciated healing of surgical excision.

## Discussion

There is no consensus about how to process melanocytic lesions. The AJCC Cancer Staging Manual does not discuss this topic [5]. The Royal College of Pathologists recommended the examination of the whole lesion [6]. Only some studies have made a thorough histological examination of the entire lesion after complete surgical excisions [7]. The case presented here is an example of a large AML with focal invasiveness which could only be identified by complete histological analyses.

It was suggested that pathologists should not hesitate to make AML diagnosis when dealing with an atypical proliferation of melanocytes along the dermal-epidermal junction in a flat, irregular shaped, darkely pigmented lesion bigger than 7 mm [1].

Tumour thickness (Breslow depth), male gender, and amelanosis are independent clinical prognostic factors for both ALM-specific and disease-free survival [3]. High mitotic rate (>6 mitoses/mm^2^) and presence of microsatellites are other important independent indicators of the metastatic potential of ALM and prognosis [4]. In this case, the patient attends clinical follow-up and a good prognosis is expected. For this case, it is important to highlight that without a complete histological examination of the surgical specimen, the patient could be sub- staged. In only three of fifty-three slides, the invasive component was identified. The exact place of the invasion could not be seen by clinical, dermatoscopy or grossing evaluation; only in the histological analysis it was identified.

## Conclusion

Even large melanocytic skin specimen, such as the presented case, sectioning through the entire lesion is very important to help determine the appropriate stage of the disease and the correct treatment and follow-up of the patient.

## Consent

Written informed consent was obtained from the patient for publication of this Case Report and any accompanying images. A copy of the written consent is available for review by the Editor-in-Chief of this jornal.
